# To the Public

**Published:** 1826-07

**Authors:** 


### Quarterly Periscope

OF

### PRACTICAL MEDICINE;

BEING

### The Spirit of the Medical Journals,

*Foreign and Domestic;*

WITH COMMENTARIES.

### TO THE PUBLIC.

Time has only confirmed us in our anticipations respecting the importance of this department of our Journal. Every year we have been obliged to enlarge its boundaries—for this good reason, that every year has multiplied the sources whence its materials spring. Periodical publications in literature, arts, sciences, and medicine, appear, like Aaron's serpent, to be swallowing up all other modes of circulating knowledge. But this multiplicity of Medical Journals, unavoidably brings into the market a prodigious glut of unprofitable wares, which imperiously demand an eclectic Periscope, for the purpose of separating the wheat from the chaff. The labour is great, but the results are of vital advantage to the general body of practitioners, no individual of whom, could possibly come at such a concentrated mass of information through any other channel than that which we have constructed. The utility—the absolute necessity, of such a department is now so universally acknowledged, that we need say nothing farther on that point—but we deem it only bare justice to ourselves, to state that we give, in each Number of our Journal, between 60 and 80 pages of letter-press, in small type, (a moderate volume in the year) beyond the ratio and proportion of price, as charged by other Journals, quarterly or monthly. This sacrifice we cheerfully make, and beg it to be considered, not as a mark of generosity, but of gratitude. The patronage of the public has enabled the Journal to bear this increase of expense, and it has stimulated the conductors of the Periscope, to willingly undergo this addition of labour.

From Europe and America, new Medical Journals are daily pouring in upon us, and, notwithstanding all the latitude which we have given to this department of our work, we find it difficult—sometimes impossible to keep even pace with the tide of periodical publication. This consideration, however, shall not induce us to deviate from the plan which we have hitherto pursued—that of making each Periscopic article complete in itself, rather than that of spreading before the reader a longer list of titles, and leaving him to seek satisfactory information in sources beyond his reach.

In our commentaries, it has been our object to benefit the reader without hurting the feelings of the writer—and the very few reclamations which have been made on our conduct, sufficiently prove that we have not been unsuccessful in this line of policy.

We beg, in conclusion, to inform our readers, that the extent of the Periscope, renders it necessary to commit that department to press about the middle of the quarter, in consequence of which, its *classification* under the various heads of physiology, pathology, &c. is unavoidably transferred to the table of contents, in order that we may be unfettered by an arrangement of this kind, while working off the Periscope in the course of the quarter. By this plan, the reader has all the advantages of the classification, while the conductors have an increase of facility in the management of the press.

